# *Styela clava*-Derived Peptide Cla-H Is a Promising Green Biofungicide for Crop Protection

**DOI:** 10.3390/biom16091326

**Published:** 2026-09-11

**Authors:** Yuliang Xu, Lang Su, Yating Zhang, Changfu Li, Yansheng Zhang

**Affiliations:** Shanghai Key Laboratory of Bio-Energy Crops, School of Life Sciences, Shanghai University, Shanghai 200444, China; xyltta0ssaw@shu.edu.cn (Y.X.); sulang@shu.edu.cn (L.S.); zyating@shu.edu.cn (Y.Z.)

**Keywords:** antimicrobial peptide, *Fusarium graminearum*, *Botrytis cinerea*, yeast fermentation

## Abstract

**Background**: Crop fungal diseases cause huge yield losses globally, and the abuse of chemical fungicides has led to severe resistance and environmental risks. Antimicrobial peptides (AMPs) represent ideal green alternatives, but poor salt tolerance and high production costs limit their field application. **Results**: In this study, we developed a His-tag engineering strategy to enhance salt tolerance of antifungal peptides, and identified a novel peptide Cla-H derived from *Styela clava*. Cla-H was efficiently expressed via secretory fermentation in *Pichia pastoris*, and its crude fermentation supernatant could be directly applied to plants. Cla-H exhibited strong and rapid fungicidal activity against *Fusarium graminearum* and *Botrytis cinerea*, accompanied by irreversible inhibition of spore germination and observable membrane permeabilization phenotypes. While SYTOX green and PI staining indicated membrane-damaging events, adequate control experiments are still required to firmly establish membrane permeabilization as its primary mode of action. Meanwhile, it showed outstanding thermal and storage stability. Most importantly, His-tag modification significantly improved its salt tolerance, enabling stable activity under high-salt conditions that completely inactivated the parental peptide. In planta assays demonstrated that crude Cla-H fermentation supernatant effectively controlled wheat scab and tomato gray mold. **Conclusions**: This study provides a promising salt-tolerant antifungal peptide for crop protection and establishes a simple and low-cost technical route for developing next-generation green biofungicides.

## 1. Introduction

Crop plants are perpetually threatened by fungal and oomycete pathogens, which inflict severe pre- and post-harvest yield losses [1,2,3]. Fungal pathogens account for 10–23% of yield losses in major crops, with post-harvest losses reaching nearly 10% [3,4]. Current crop protection relies heavily on synthetic fungicides [5]. Most of these chemistries interact with one specific enzyme or protein receptor, rather than triggering multi-target damage. Due to this single-site mode-of-action, pathogens readily generate target-site mutations under persistent chemical pressure, driving the rapid evolution of fungicide-resistant populations [6]. Consequently, the efficacy of many fungicides gradually declines, and their residues bring considerable ecological risk [7]. For instance, quinone outside inhibitor (QoI, strobilurin-type) fungicides specifically suppress fungal respiration by targeting mitochondrial cytochrome b. A single amino-acid substitution (G143A) in cytochrome b abolishes fungicide binding. Repeated fungicide exposure has driven widespread QoI-resistant field populations in diverse plant-pathogenic fungi [8]. Therefore, there is an urgent need for developing new antifungal agents with unique modes of action, high efficiency and eco-friendliness.

Antimicrobial peptides (AMPs) are ubiquitous core defensive molecules found across diverse organisms, including plants, marine animals, and microorganisms, and have emerged as promising molecular candidates for novel fungicide development [8,9,10]. Distinct from traditional chemical fungicides, most AMPs exert antifungal effects via multi-site modes of action (MoAs), which effectively reduces the risk of pathogen resistance evolution, endowing them with unique advantages for agricultural disease control. Plant-derived AMPs are among the most well-characterized natural antifungal peptides. As typical cysteine-rich cationic AMPs, defensins feature a conserved cysteine-stabilized α/β (CSαβ) motif and exhibit strong broad-spectrum antifungal activity [11,12,13,14]. The model plant defensin MtDef4, derived from *Medicago truncatula*, inhibits *Fusarium graminearum* infection via four distinct mechanisms, including membrane permeabilization, phosphatidic acid binding, cell internalization and intracellular localization [15,16,17]. Its truncated 17-amino-acid peptide GMA4CG_V6, originating from the γ-core motif of MtDef4, displays prominent inhibitory activity against *Botrytis cinerea* and possesses superior salt tolerance compared with the full-length MtDef4 [18]. ZmD32, a functional AMP isolated from Zea mays, also exhibits considerable antifungal potential for crop disease control [19]. In addition to plant sources, marine animal-derived AMPs constitute another valuable reservoir of antifungal agents for agricultural application. Multiple peptides derived from marine animals have been confirmed to exhibit favorable antimicrobial properties, among which Clavanin AK from *Styela clava* [20] and Ci-MAM-A24 from *Ciona intestinalis* [21] show prominent inhibitory activity against pathogenic bacteria. Apart from natural AMPs, artificially synthetic antifungal peptides also serve as important candidates for novel fungicide development, such as the synthesized peptide HnMc-WP2 [22]. Despite the excellent antimicrobial efficacy of the above natural and synthetic AMPs, the majority are sensitive to high-ionic-strength conditions. Such susceptibility compromises their bioactivity under the ionic environment and therefore hinders their large-scale deployment for plant disease management in agriculture.

Direct application of microbial fermentation broth containing cationic AMPs is restricted, as high ion concentrations in the broth impair the bioactivity of AMPs [19,23]. Complex downstream purification further increases production cost, making large-scale application economically unviable. To promote the practical application of AMPs in agriculture, it is necessary to develop molecular modification strategies that simultaneously enhance salt tolerance and enable efficient expression in microbial chassis.

Histidine tagging has been proposed as a promising engineering approach to enhance peptide salt tolerance [24,25]. It can increase net positive charge of peptides, weaken salt-induced activity interference, and simplify subsequent purification. Nevertheless, whether His-tagging represents a general salt tolerance engineering strategy remains poorly validated, since the functional outcome is strongly shaped by the intrinsic physicochemical features of individual peptides. High ionic strength occurs both within *Pichia pastoris* fermentation broths and on plant leaf surfaces under field conditions, which readily inactivates many cationic AMPs and constitutes a major barrier to direct crude-broth application. In this study, we designed ten His-tagged variants based on five native AMPs, expressed them heterologously in *Pichia pastoris*, and screened variants with good salt tolerance and stable antifungal activity. The selected Cla-H was systematically characterized for antifungal potency, stability and plant protection effect. This study identifies an antifungal peptide effective against *F. graminearum* and *B. cinerea*. With markedly enhanced salt tolerance, its yeast fermentation product can be directly applied for plant fungal disease control, providing a straightforward approach for developing eco-friendly antifungal agents.

## 2. Materials and Methods

### 2.1. Fungal Strains and Culture Conditions

*F. graminearum* (strain BNCC342051) and *B. cinerea* Pers. (strain BNCC123731) were obtained from the BeNa Culture Collection, China. Both strains were routinely cultured on PDA medium (20% potato infusion, 2% glucose, 1.5% agar) at 25 °C. For spore preparation, *F. graminearum* was cultured for 7–14 d and *B. cinerea* for 11–25 d on PDA plates. Spores were eluted with sterile 0.85% NaCl containing 0.05% Tween 20, filtered to remove mycelia, centrifuged at 1500× *g* for 5 min, and re-suspended in synthetic low-salt fungal medium (SFM) (2.5 mM KHPO_4_, 50 μM MgSO_4_, 50 μM CaCl_2_, 5 μM FeSO_4_, 0.1 μM CoCl_2_, 0.1μM CuSO_4_, 2 μM NaMoO_4_, 0.5 μM HBO_3_, 0.1 μM KI, 0.5 μM ZnSO_4_, 0.1μM MnSO_4_, 10 g/L glucose, 1 g/L asparagine, 20 mg/L methionine, 2 mg/L myo-inositol, 0.2 mg/L biotin, 1 mg/L thiamine-HCl, and 0.2 mg/L pyridoxine-HCl) for subsequent activity determination.

### 2.2. Construction of the Yeast Strains Expressing the Peptides of Interest

Five parental AMPs, namely Ci-MAM-A24 (Ci), GMA4CG_V6 (GM), ZmD32 (Zm), HnMc-WP2 (Hn), and Clavanin AK (Cla), served as templates. Their biological origins, amino-acid sequences, molecular weights, isoelectric points and net charges are summarized in Table S1. Ten His-tagged variants were designed by adding a 6 × His-tag at either the N or C terminus. The coding sequences of the 10 His-tagged peptides were cloned into the *P. pastoris* expression vector pPIC9K, downstream of the α factor secretion signal. Recombinant plasmids were linearized with SacI and electroporated into *P. pastoris* GS115. Transformants were selected on MD plates (1.34% YNB, 4 × 10^−5^% biotin, 2% dextrose). High-copy integrants were screened using gradient G418 resistance (0.25 to 4.0 mg/mL). Positive clones were confirmed by genomic PCR using AOX1-specific primers.

### 2.3. Yeast Fermentation

Ten fresh colonies were inoculated into BMGY medium (1% yeast extract, 2% peptone, 1.34% YNB, 4 × 10^−5^% biotin, 1% glycerol, 100 mM potassium phosphate pH 6.0) and grown at 30 °C, 220 rpm to OD_600_ = 6–8. Cells were harvested and re-suspended in BMMY medium (identical to BMGY but with 1% methanol instead of glycerol) to OD_600_ = 1.0 and induced at 28 °C for 96 h, with 0.5% methanol added every 24 h. Culture supernatants were collected by centrifugation at 12,000× *g* for 15 min at 4 °C and stored at −80 °C.

### 2.4. Chemical Synthesis and Purification of the Peptides

Clavanin AK-His (Cla-H), as well as its parent form (Cla), was chemically synthesized via solid-phase peptide synthesis by Biopepmic Co., Ltd. (Suzhou, China), with a purity higher than 95%. Peptides were purified using reverse-phase high-performance liquid chromatography (RP-HPLC) on a Shimadzu inertsil ODS-SP C18 column (4.6 mm × 250 mm, 5 μm). The RP-HPLC conditions were set as follows: mobile phase A consisted of 0.1% trifluoroacetic acid (TFA) in ultrapure water, and mobile phase B was 0.1% TFA in acetonitrile, the total flow rate was 1 mL/min, and the detection wavelength was 220 nm. The purified peptides were further identified by mass spectrometry.

### 2.5. Antifungal Activity Assays

Antifungal activity was evaluated via the broth microdilution assay with resazurin staining. All reaction systems had a final volume of 100 μL and were prepared by mixing double-concentration spore suspension in PDB with various test solutions at a 1:1 (*v*/*v*) ratio. The tested solutions included BMMY medium (pH 6.0), supernatant from the empty-vector-integrated strain, and fermentation supernatants of recombinant peptide-expressing strains. Assays were performed at three spore concentrations: 10^4^, 10^5^, and 10^6^ spores/mL.

For determining the minimum inhibitory concentration (MIC) of purified peptides, 50 μL peptide solution was mixed with 50 μL SFM containing fungal spores at a concentration of 5 × 10^4^ spores/mL in 96-well plates. All plates were incubated at 25 °C for 48 h. Afterwards, 0.01% resazurin solution was added, and incubation continued for another 2–4 h. The MIC was defined as the lowest peptide concentration that showed no sign of blue-to-pink color transition of resazurin. For salt tolerance assays, SFM was supplemented with 200 mM KCl prior to use to achieve a final KCl concentration of 100 mM in the assay system.

### 2.6. Spore Germination Inhibition Assay

Spores (1 × 10^6^ spores/mL) were treated with peptides at MIC for 1, 2 or 6 h, washed twice with SFM to remove unbound peptides, and incubated in SFM at 25 °C. Experiments were performed with three independent biological replicates. Germination rates were calculated by counting ≥50 spores per treatment.

### 2.7. Killing Kinetics and Propidium Iodide (PI) Staining

Spores were treated with peptides at MIC for 1, 2 or 4 h, then stained with 0.5 μg/mL PI for 10 min in the dark. Samples were observed under a fluorescence microscope. Experiments were performed with three independent biological replicates. The percentage of PI-positive (dead) spores was calculated from ≥50 spores per sample.

### 2.8. Membrane Permeabilization Assay with SYTOX Green

Spores or germlings were incubated with peptides at MIC for 2 h, then stained with 1 μM SYTOX green for 15 min. Fluorescence was observed by fluorescence microscopy (excitation 488 nm, emission 505–550 nm). The percentage of fluorescent hyphae or spores was quantified as a measure of membrane damage.

### 2.9. Thermal Stability Assay

Peptides were incubated at 25, 50, 75 or 90 °C for 30 min, or stored at 25 °C for 7 days. Residual antifungal activity was determined by MIC assays as described above.

### 2.10. In Planta Antifungal Bioassay

In this study, the commonly used susceptible wheat cultivar *Triticum aestivum* cv. Jimai 22 and tomato cultivar *Solanum lycopersicum* cv. Ailsa Craig were adopted for plant infection assays. All plant materials were grown in a programmable growth chamber with controlled conditions: a 16 h light/8 h dark photoperiod, day/night temperatures of 25 °C/20 °C, and 70% relative humidity. Ten- to fifteen-day-old wheat seedlings and four-week-old tomato plants were pre-treated with crude fermentation supernatants containing target peptides via foliar spraying. Plants sprayed with the fermentation supernatant from empty-vector-transformed strain served as the negative control group. For wheat plants, each treatment included three biological replicates (25–30 seedlings per replicate), and disease incidence was calculated as the percentage of symptomatic seedlings within each replicate. For tomato, three independent plants were included per treatment, and disease incidence was assessed by enumerating symptomatic leaves. After a 2 h interval, all plants were inoculated with fungal spore suspensions at a concentration of 1 × 10^6^ spores/mL. Disease incidence was evaluated at 30 days post inoculation for wheat seedlings and 10 days post inoculation for tomato.

### 2.11. Statistical Analysis

All experiments were performed in triplicate. Data were analyzed by ONE-WAY ANOVA with Tukey’s post hoc test using GraphPad Prism 8.0. Differences were considered statistically significant at *p* < 0.05, with significance levels denoted as * *p* < 0.05, ** *p* < 0.01, *** *p* < 0.001, and **** *p* < 0.0001.

## 3. Results

### 3.1. Design and Construction of His-Tagged Antifungal Peptide Variants

Five parent antimicrobial peptides (Ci, GM, Zm, Hn, and Cla; their full names are provided in Section 2) were screened and chosen for investigation. Detailed information of these peptides is summarized in Table S1. Ten modified derivatives were constructed via N- or C-terminal fusion with a 6 × His-tag, yielding the recombinant peptides Ci-H, H-Ci, GM-H, H-GM, Zm-H, H-Zm, Hn-H, H-Hn, Cla-H and H-Cla. All coding sequences were inserted into the *P. pastoris* secretory expression vector pPIC9K, between the double EA repeats coding sequence and the *NotI* restriction site, and integrated into the genome of strain GS115 via electroporation. Positive recombinant strains were verified by PCR of their genomic DNAs (Figure S1). A panel of engineered yeast strains was successfully generated, harboring either the empty vector or distinct peptide-encoding inserts, designated G-pPIC9K, G-Ci-H, G-H-Ci, G-GM-H, G-H-GM, G-Zm-H, G-H-Zm, G-H-Hn, G-Hn-H, G-Cla-H and G-H-Cla.

During preliminary shake-flask fermentation, two peptide variants, H-Zm and H-Hn, exerted severe growth-inhibitory effects on *Pichia pastoris*, which indicated prominent cytotoxicity. Consequently, the two constructs were eliminated from subsequent experimentation. The remaining eight peptide variants were successfully secreted into the culture supernatants, as confirmed by Tricine-SDS-PAGE (Figure S2).

To further verify peptide biosynthesis, we carried out HPLC detection for the secreted Cla-H peptide, with chemically synthesized and purified Cla-H serving as a reference standard. Compared with the fermentation supernatant of the empty-vector transformant G-pPIC9K, a new peak was observed in the supernatant from the engineered strain G-Cla-H. Nevertheless, its retention time (5.5 min) differed substantially from that of the synthetic Cla-H standard (13.15 min) (Figure S3). We then harvested and purified this new peak for mass-spectrometric analysis. Its ion-fragment profile perfectly matched that of chemically synthesized Cla-H (Figure S4). These findings demonstrate that the yeast strain G-Cla-H synthesizes and secretes Cla-H into the fermentation supernatant. The discrepancy in HPLC retention time is most likely caused by differing secondary-structural conformations between biosynthesized and chemically synthesized Cla-H. We quantified secreted Cla-H based on the synthetic Cla-H standard, and the shake-flask titer reached about 156 mg/L.

### 3.2. Screening of Fermentation Supernatants Identifies Two Potent Peptides

**Figure 1:**
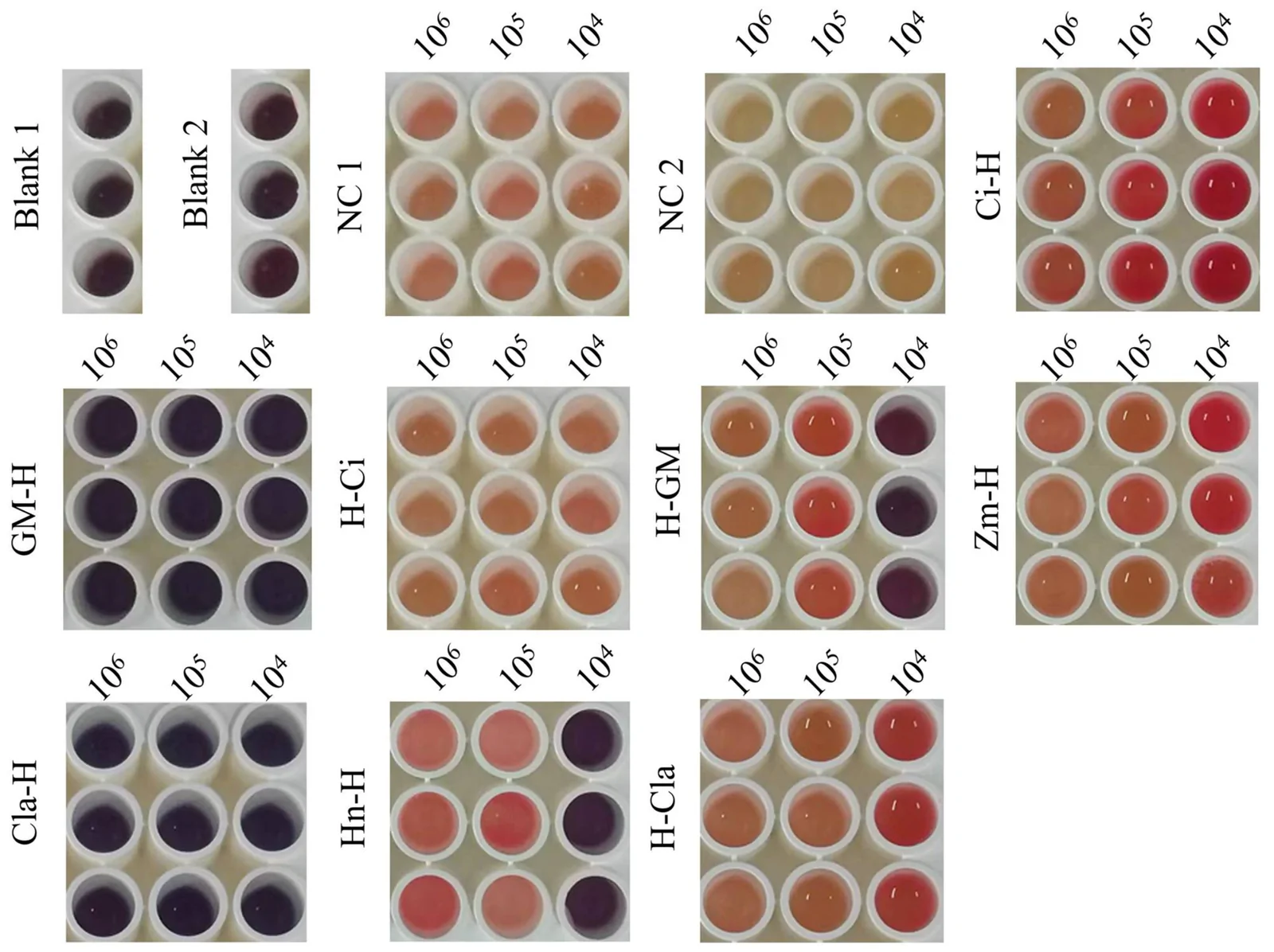
Antifungal activity of fermentation supernatants from recombinant *P. pastoris* strains against *F. graminearum* determined using a resazurin colorimetric assay. All reaction systems had a final volume of 100 μL. Each system was prepared by mixing double-concentration spore suspension in PDB with an equal volume of test solutions at 1:1 (*v*/*v*) ratio. Two blank controls were set up: Blank 1 consisted of PDB mixed with BMMY medium; Blank 2 was prepared by combining PDB with supernatant from the empty-vector-integrated strain. Two negative controls were also included: Negative Control 1 (NC 1) contained BMMY and *F. graminearum* spores in PDB; Negative Control 2 (NC 2) contained supernatant from the empty-vector-integrated strain and *F. graminearum* spores in PDB. Each treatment group was composed of fermentation supernatant from the corresponding recombinant strain and *F. graminearum* spores in PDB. Columns marked with 10^6^, 10^5^, and 10^4^ indicate tests conducted with spore concentrations of 10^6^, 10^5^, and 10^4^ spores mL^−1^, respectively. The abbreviations beside each nine-well panel represent different engineered antimicrobial peptide variants.

The antifungal activities of shake-flask fermentation supernatants against *F. graminearum* (Figure 1) and *B. cinerea* (Figure S5) were screened via the resazurin-based microdilution assay, with fungal spore gradients of 10^4^, 10^5^ and 10^6^ spores·mL^−1^ set for each test group. As observed from the 96-well-plate results, the spore-free Blank 1 (BMMY medium mixed with resazurin) and Blank 2 (supernatant from empty-vector-transformed yeast strains together with resazurin) retained an intense deep-purple color. This result confirmed that neither BMMY medium nor the yeast fermentation supernatant could reduce resazurin. In contrast, prominent color fading occurred in the Negative Control 1 (NC 1: BMMY medium supplemented with resazurin and fungal spores) and the Negative Control 2 (NC 2: supernatant harvested from the empty-vector transformant mixed with resazurin and fungal spores). This color change confirms that the blank medium and empty-vector yeast supernatant possesses no intrinsic fungistatic activity, and viable fungal spores account for the reduction reaction of resazurin. All of these control-group findings validated the credibility of our resazurin microdilution detection system for antifungal screening.

Eight secreted 6 × His-tagged peptide variants were subsequently assessed. Most candidates, including Ci-H, H-Ci, H-GM, Zm-H, Hn-H and H-Cla, displayed bright-red well coloration across the spore-density gradient, which represented vigorous fungal growth. Only GM-H and Cla-H maintained deep-purple color within all sample wells over 10^4^–10^6^ spores/mL treatments (Figure 1 and Figure S5), suggestive of their strong and reproducible inhibitory capacity against both phytopathogenic fungi under salt-supplemented fermentation conditions.

**Figure 2:**
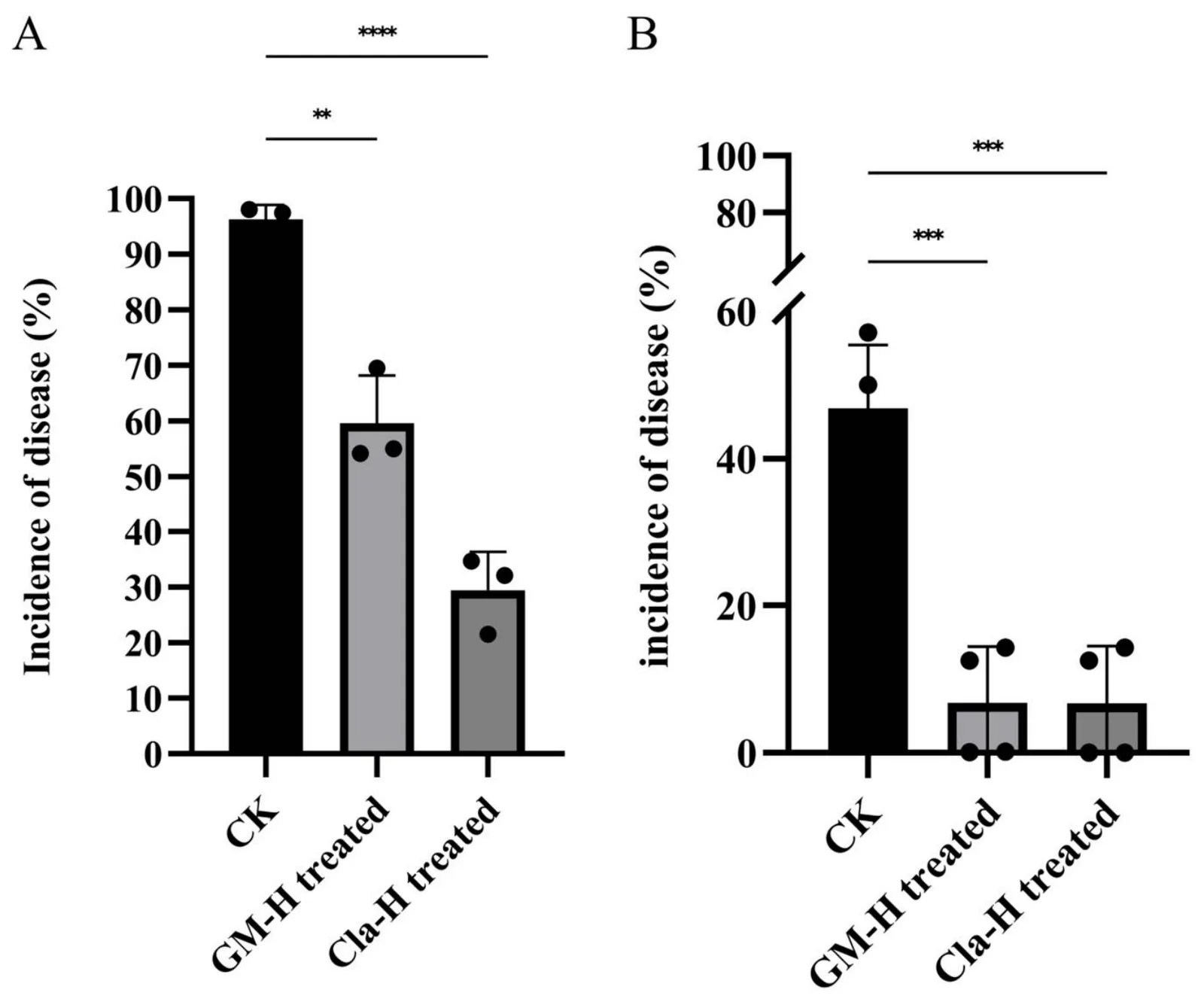
In vivo antifungal efficacy of fermentation supernatants containing GM-H and Cla-H. Disease incidence was determined for wheat plants infected with *F. graminearum* (**A**) and tomato plants infected with *B. cinerea* (**B**). Differences were considered statistically significant at *p* < 0.05. Significance levels were denoted as ** *p* < 0.01, *** *p* < 0.001, and **** *p* < 0.0001.

The fermentation supernatants containing GM-H and Cla-H were then further assessed for antifungal activity through in vivo plant assays. Wheat seedlings and tomato plants were separately sprayed with crude supernatants containing GM-H or Cla-H, prior to inoculation with the corresponding pathogenic fungi. For wheat seedlings infected with *F. graminearum*, the disease incidence was 96.3 ± 2.6% in the control group sprayed with fermentation supernatant of the empty-vector-transformed yeast strain. By comparison, the incidence decreased to 59.6 ± 8.6% and 29.5 ± 7.0% in seedlings treated with GM-H and Cla-H supernatants, respectively (Figure 2A). In tomato seedlings challenged with *B. cinerea*, the control group showed a disease incidence of 46.9 ± 8.5%. Treatment with either peptide-containing supernatant lowered the incidence to below 7.0% (Figure 2B). Representative images of the treated and control plants are displayed in Figures S6 and S7.

These findings indicate that the crude fermentation supernatant containing GM-H or Cla-H is applicable for direct disease control. Although the antifungal capacity of the parental peptide GM (the precursor of GM-H) against *B. cinerea* has been documented in a previous report [18], Cla-H is newly identified to inhibit both *F. graminearum* and *B. cinerea*. Accordingly, we selected Cla-H for subsequent biochemical and functional characterization.

### 3.3. Cla-H Retains Potent Antifungal Activity and Exhibits Greatly Improved Salt Tolerance

To further evaluate the antifungal capacity of Cla-H, we measured its MIC using a resazurin-based microdilution assay. It should be noted that exact MIC values can fluctuate among independent plate incubations due to variable culture environments and differences in spore viability; however, intra-run MIC comparisons between different peptides are trustworthy. We thus performed side-by-side testing of biosynthesized Cla-H purified from G-Cla-H fermentation supernatant and its chemically synthesized counterpart against *F. graminearum* and *B. cinerea* under low- and high-salt conditions within one experimental batch. Biosynthesized and synthetic Cla-H showed equivalent inhibitory potency against both phytopathogens at each salinity level (Figures S8 and S9).

To further explore how the C-terminal His-tag fusion influences the antifungal activity of Cla, we chemically synthesized the parental peptide Cla and carried out parallel antifungal comparison between synthetic Cla and Cla-H under low- and high-salt conditions. Under low-salt conditions, synthetic Cla and Cla-H displayed comparable bioactivity. Both presented an MIC of 3.125 μM against *F. graminearum* and 12.5 μM against *B. cinerea* (Table 1). This finding confirmed that C-terminal 6 × His-tag modification does not compromise the intrinsic antifungal potency of the native Cla peptide. Upon high-salt stress, Cla completely lost detectable inhibitory activity against the two phytopathogens. In contrast, Cla-H retained strong antifungal capacity, with MIC values fixed at 25 μM for both pathogens (Table 1).

The 100 mM KCl high-salt condition used in this in vitro assay recapitulates the elevated ionic strength encountered in *P. pastoris* culture supernatants, as well as salt accumulation that can occur on plant leaf surfaces under field conditions. The retained activity of Cla-H under this ionic environment provides a biochemical basis supporting the direct use of unpurified fermentation supernatant for plant disease control. By contrast, the parental peptide Cla lost activity under such ionic loads, which would preclude its crude-broth application in practical agricultural scenarios.

We also evaluated the in vitro antifungal potency of the commercial fungicide ‘Jin-Mai-Tian’ against *F. graminearum* via microdilution MIC assay under low-ionic-strength culture conditions. As a widely used agent for wheat scab management manufactured by Syngenta, this formulated fungicide contains two fungicidal components, pydiflumetofen (Py) and propiconazole (Pr). MIC bioassays revealed that the chemically synthesized Cla-H yielded an MIC value of 3.125 μM and exhibited markedly better antifungal performance than this commercial agrochemical, whose MIC was 6.63 μM for pydiflumetofen (Py) combined with 5.63 μM for propiconazole (Pr) (Figure S12).

### 3.4. Cla-H Rapidly and Irreversibly Inhibits Spore Germination

**Figure 3:**
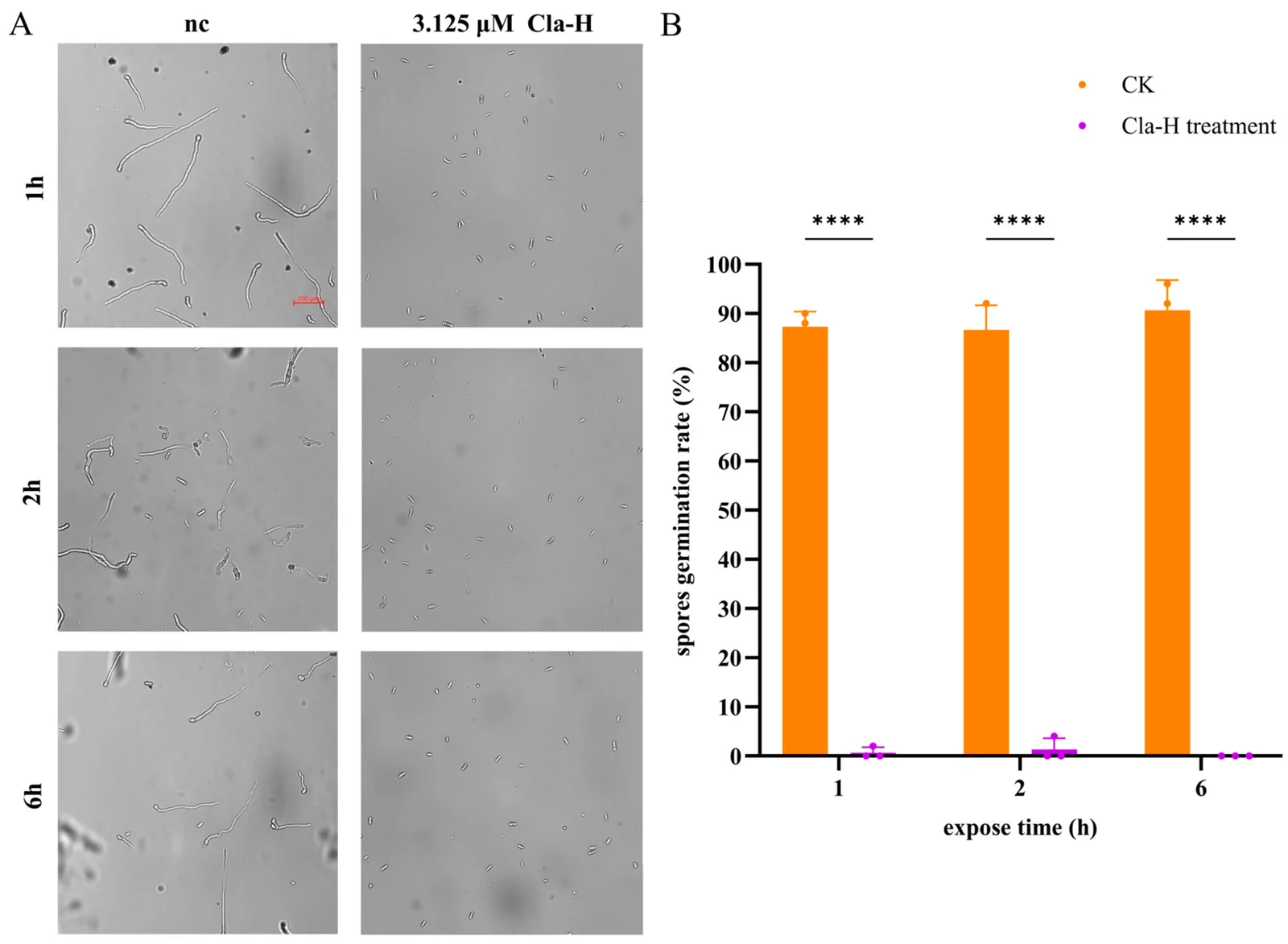
Inhibitory effects of Cla-H on spore germination of *F. graminearum*. Spores were incubated with Cla-H at MIC of 3.125 μM for 1, 2 or 6 h, followed by washing to eliminate unbound peptide. Panel (**A**) shows representative microscopic images of spore morphology, and panel (**B**) presents statistical data of germination inhibition ratios. Differences were considered statistically significant at *p* < 0.05. Significance levels were denoted as **** *p* < 0.0001.

Fungal spores were exposed to 3.125 μM Cla-H (the corresponding MIC) for 1, 2 and 6 h, followed by thorough washing to remove unbound peptides. Microscopic observation showed that nearly all treated spores of *F. graminearum* (Figure 3) and *B. cinerea* (Figure S13) failed to germinate, with no germ tube formation or polarized growth. The results proved that 1 h treatment was enough to cause irreversible damage to fungal spores.

### 3.5. Cla-H Impairs the Integrity of Fungal Cell Membranes

**Figure 4:**
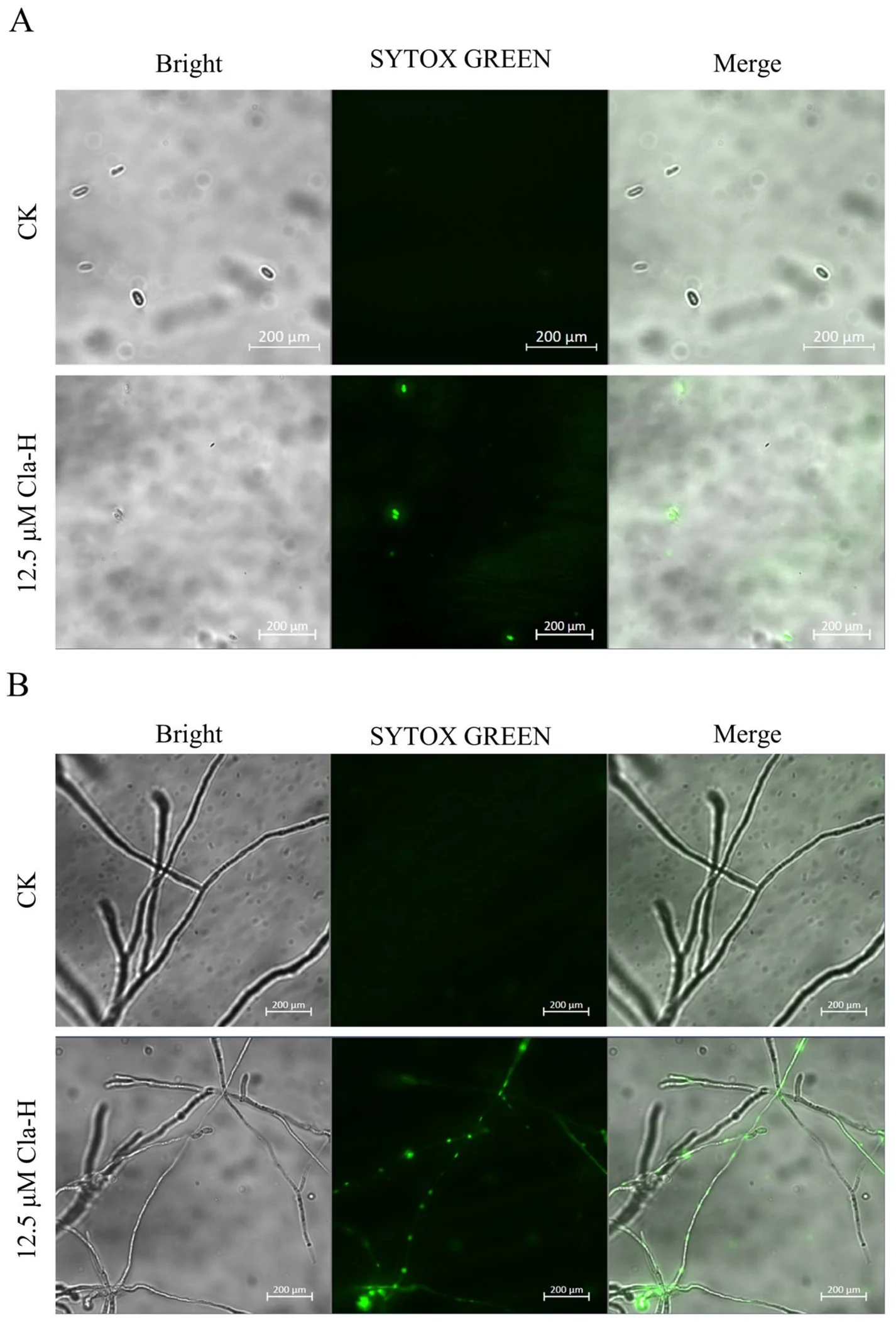
SYTOX green fluorescence micrographs showing elevated plasma membrane permeability spores (**A**) and hyphae (**B**) of *F. graminearum* after Cla-H treatment. Images were captured under bright-field, SYTOX green fluorescence, and merged channels. CK: negative control, representing spores or hyphae incubated with sterile water without Cla-H treatment. Green fluorescence indicates permeabilized cells. Scale bars = 200 μm.

SYTOX green staining was performed to evaluate membrane permeabilization. To validate the reliability of this staining assay, GM-H was used as a positive control to assess its membrane-disrupting activity against *B. cinerea*, since its parental peptide GM has been previously reported to trigger substantial membrane damage in *B. cinerea* [18]. As shown in Supplementary Figure S14, GM-H induced evident membrane permeabilization in *B. cinerea*. Under this experimental setup, Cla-H caused obvious membrane damage in spores and hyphae of both *F. graminearum* (Figure 4) and *B. cinerea* (Figure S15).

**Figure 5:**
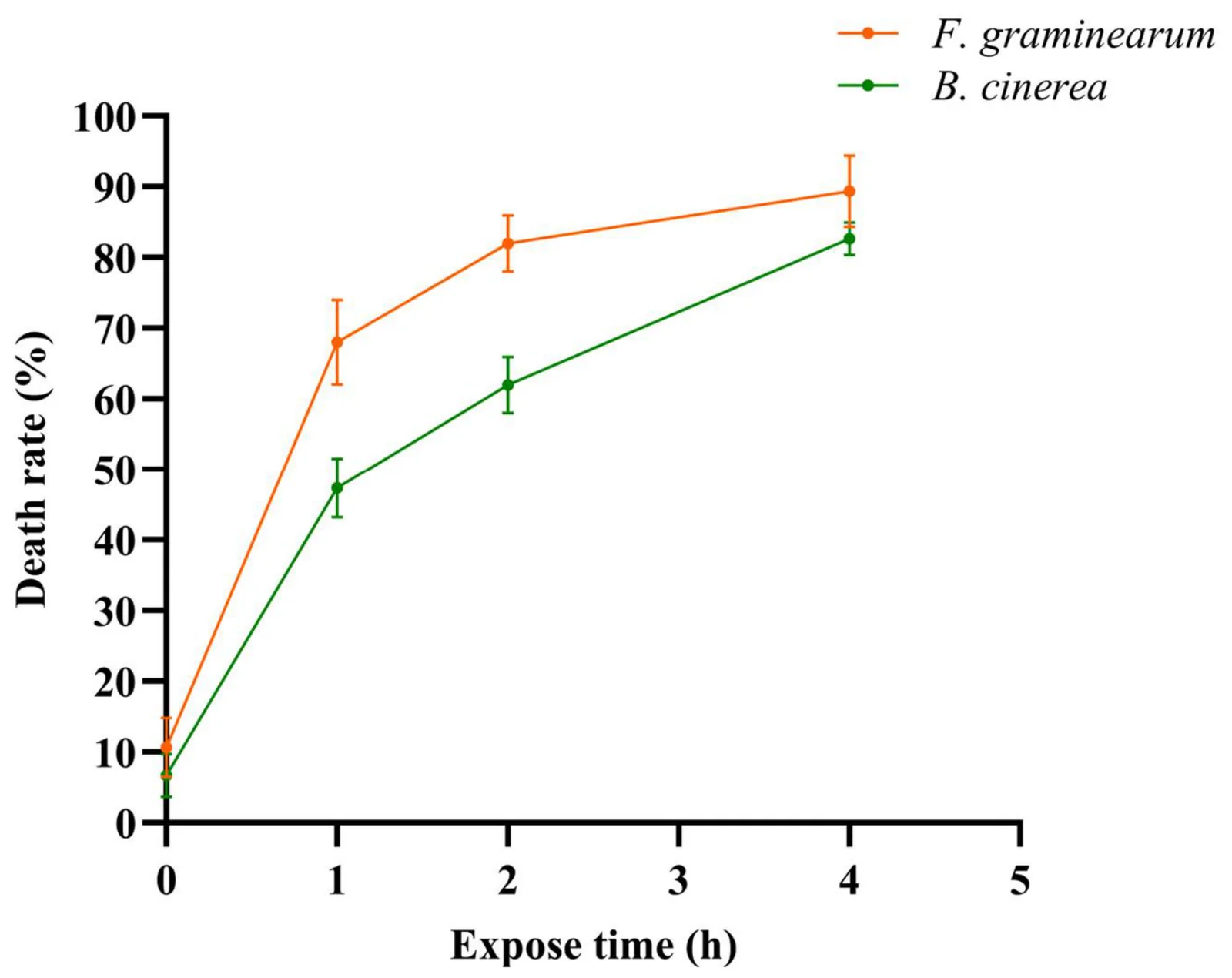
Time-dependent spore mortality of *F. graminearum* (orange line, 3.125 μM Cla-H) and *B. cinerea* (green line, 12.5 μM Cla-H) following peptide treatment, quantified via propidium iodide (PI) staining. Untreated controls at the 4 h time-point exhibited no detectable background spore mortality (Figures S15 and S16). Data are presented as mean ± SD from three independent biological replicates, with ≥50 spores counted per replicate at each time-point.

Propidium iodide (PI) staining was used to calculate spore mortality. PI-positive ratio was recorded to reflect the proportion of dead cells. For *F. graminearum*, Cla-H caused 68 ± 6.0% mortality at 1 h, 82.0 ± 4.0% at 2 h and 89.0 ± 5.0% at 4 h (orange line, Figure 5). For *B. cinerea*, Cla-H induced 47.3 ± 4.2%, 62.0 ± 4.0% and 82.7 ± 2.3% mortality at 1, 2 and 4 h, respectively (green line, Figure 5). Representative PI staining images for *F. graminearum* and *B. cinerea* are shown in Figures S16 and S17.

### 3.6. Excellent Thermal Stability Supports Field Application

**Figure 6:**
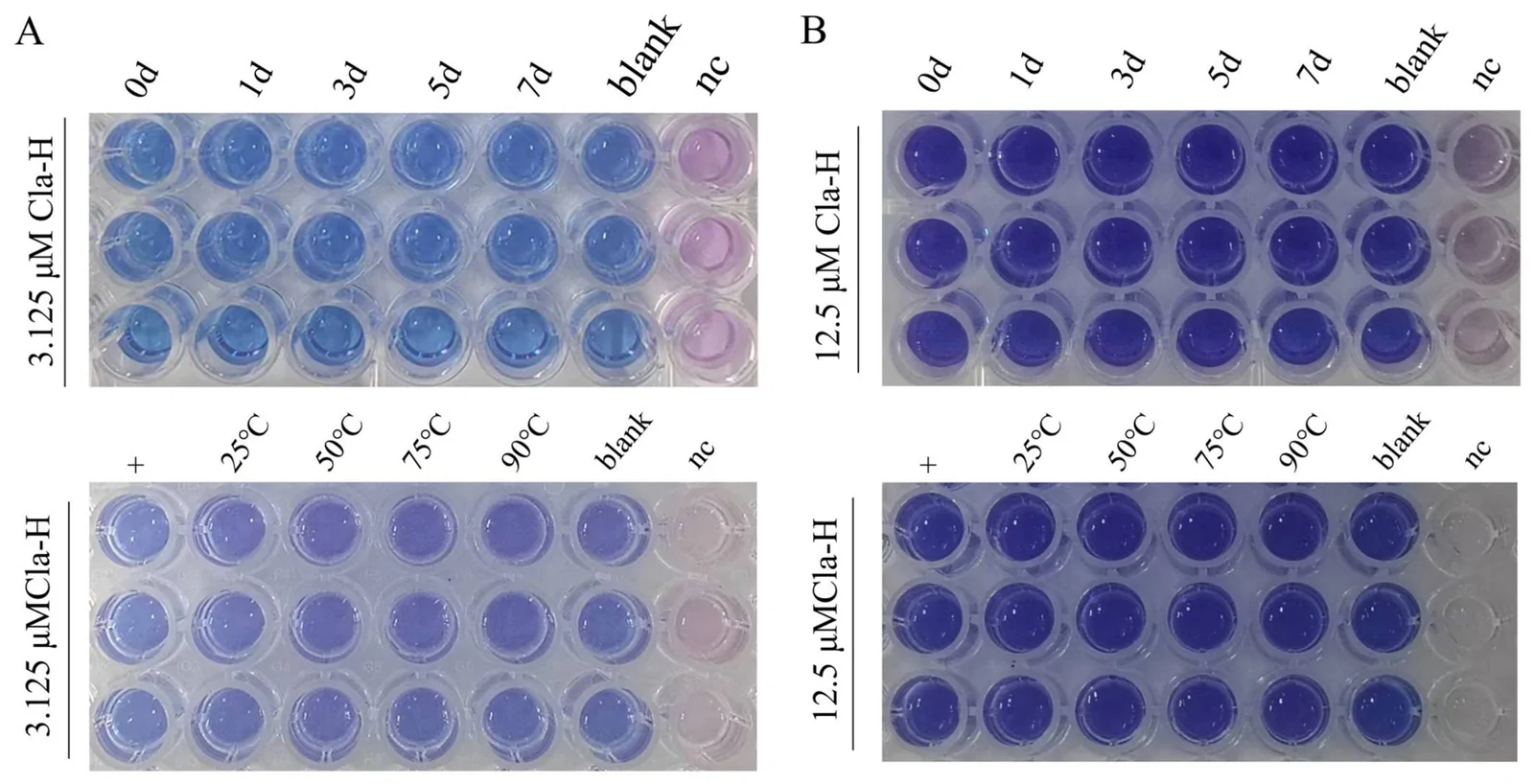
Residual antifungal activity of Cla-H against *F. graminearum* and *B. cinerea* determined by resazurin colorimetric assay. (**A**) Activity after 1–7-day storage at 25 °C; peptide concentrations: 3.125 μM (*F. graminearum*), 12.5 μM (*B. cinerea*). 0 d: initial-activity control (no pre-incubation); 1–7 d: Cla-H stored at indicated temperatures; blank: SFM + resazurin (no peptide/spores); nc: negative control (water + spores + resazurin, no Cla-H). (**B**) Activity after 30 min exposure to gradient temperatures. +: positive control (untreated Cla-H); 25–90 °C: Cla-H pre-incubated at target temperatures; blank and nc controls are defined as above.

We assessed the stability of Cla-H via two treatments: short-term heating at 25 °C, 50 °C, 75 °C and 90 °C for 30 min, and long-term storage at 25 °C for 7 days. After these two treatments, the antifungal activity of Cla-H against *F. graminearum* (Figure 6A) and *B. cinerea* (Figure 6B) showed no decline compared with the untreated control. Such excellent thermal and storage stability makes Cla-H well suited for field application, commercial production and ambient-temperature transportation.

## 4. Discussion

### 4.1. Histidine Modification Significantly Improves Salt Tolerance Without Impairing Core Activity

Native cationic AMPs mainly bind to the negatively charged fungal membrane through electrostatic interaction [26,27,28]. High concentrations of environmental cations can compete for binding sites, shield positive charge and lead to a decline in antifungal activity or complete inactivation [18,22]. Such ionic-driven inactivation presents a dual practical challenge: it occurs not only within ion-rich *P. pastoris* fermentation broths during heterologous production, but also on foliar surfaces where salts accumulate via irrigation, dew evaporation or atmospheric deposition under field conditions. Consequently, salt-sensitive AMPs cannot retain bioactivity in unpurified fermentation supernatants, and would also lose efficacy once sprayed onto crop foliage, representing a critical bottleneck limiting real-world agricultural translation. Our experimental results demonstrated that Cla-H shared nearly identical antifungal potency with its precursor peptide Cla under low-salt surroundings. This finding verifies that the C-terminal His-tag fusion does not alter the essential functional domain or native spatial conformation responsible for fungal membrane recognition. Nevertheless, obvious divergence appeared under high-salt stress (100 mM KCl): unmodified Cla suffered almost complete loss of inhibitory activity towards *F. graminearum* and *B. cinerea*, while Cla-H retained strong antifungal competence. Several molecular mechanisms could account for the relieved salt-triggered activity inhibition caused by the C-terminal histidine-rich tag. First, the His-tag increases the net positive charge of the Cla peptide, strengthening electrostatic attraction to fungal membranes and enabling Cla-H to compete with environmental cations for membrane binding under high ionic strength. Second, histidine residues dynamically stabilize peptide–membrane interactions under salt stress. They simultaneously form hydrogen bonds with lipid phosphate headgroups and coordinate ambient alkali metal ions to neutralize surface negative charges, thereby sustaining stable peptide–membrane binding and prolonged membrane residence time at high ionic strength [29,30,31].

Notably, the performance of His-tagged variants was highly dependent on the fusion position. The N-terminally modified Cla variant (H-Cla) completely lost antifungal capacity (Figure 1 and Figure S5). We infer that N-terminal fusion introduces severe steric hindrance and disrupts the spatial structure of the active region, which is critical for target recognition. This position-dependent effect is consistent with previous findings on other proteins. For instance, the N-terminal His-tag of SLPI impairs its inhibitory function by interfering with substrate binding, while C-terminal tagging causes negligible structural and functional perturbations [32]. Collectively, C-terminal His modification is a rational design for Cla, which optimizes salt tolerance without compromising its intrinsic antifungal properties.

### 4.2. Cla-H Exhibits Rapid Fungicidal Kinetics

A key merit of antimicrobial peptides over conventional fungicides is their multi-target mode of action, which strongly delays the emergence of pathogen resistance [9,11]. In this study, Cla-H displayed typical rapid fungicidal action: spore germination was irreversibly blocked after just 1 h of peptide exposure at the MIC, and treated spores remained unable to germinate even after removal of unbound peptide (Figure 3 and Figure S13). This irreversible inhibitory effect confirmed that Cla-H exerts rapid, permanent damage to fungal spores rather than transient growth suppression. Membrane permeabilization assays using SYTOX green staining revealed that Cla-H disrupts membrane integrity in both spores and hyphae of *F. graminearum* (Figure 4) and *B. cinerea* (Figure S15). Propidium iodide (PI) staining further quantified the fungicidal kinetics: spore mortality reached 68% within 1 h and rose to 89% at 4 h for *F. graminearum*, while for *B. cinerea*, mortality reached 47% at 1 h and 82.7% at 4 h (Figure 5). These results demonstrate that Cla-H kills fungal pathogens quickly by destroying cell membrane integrity, consistent with the membrane-disruptive mechanism widely observed in cationic antifungal peptides. Nevertheless, because these permeabilization assays were performed without positive and negative controls (e.g., a known membrane-lytic peptide as a positive reference and an inactive peptide variant as a negative control), the present data should be regarded as suggestive rather than definitive. They support—but do not yet establish—membrane permeabilization as the primary mode of action, and we therefore refrain from an unequivocal mechanistic conclusion until such controls are incorporated in follow-up studies. Notably, Cla-H showed clear pathogen-specific action patterns. For *F. graminearum*, it permeabilized both spores and hyphae; for *B. cinerea*, it also targeted both developmental stages but with lower early-time mortality. Such specificity suggests that Cla-H recognizes pathogen-specific membrane components or surface charges, enabling precise and efficient antifungal action while reducing non-specific interactions.

### 4.3. Thermal Stability and Crude Application Lay the Foundation for Practical Deployment

Stability is an important index for industrial production and field promotion of AMPs. Cla-H maintained complete activity after 90 °C treatment for 30 min, and stable after 7 d storage at 25 °C (Figure 6). Such characteristics enable the peptides to tolerate high-temperature processing, protease degradation and long-term storage, adapting to commercial preparation and field spray application. It is worth noting that thermal and storage stability were only determined for purified Cla-H in the current work. Additional stability assessments of crude fermentation supernatant under practical agricultural conditions are needed to verify its direct-application potential for crop protection. More importantly, unpurified fermentation supernatant showed excellent control effect on wheat scab and tomato gray mold. Direct application of crude supernatant avoids affinity chromatography, ion exchange and other complex purification steps, greatly lowering production cost and facilitating large-scale field popularization.

Notably, the Cla-H-containing crude supernatant holds great promise as an eco-friendly biofungicide amenable to direct field application; nevertheless, multiple critical practical obstacles restricting its on-site deployment have yet to be resolved. Comprehensive evidence concerning batch-to-batch consistency of the crude supernatant, residue persistence on plant leaf surfaces after spraying, and adverse impacts upon non-target organisms is still lacking. To sufficiently justify the feasibility of direct field utilization and systematically clarify its ecological safety, further research is required to explore influences of Cla-H on beneficial soil bacteria, plant endophytes and other non-target microorganisms, since its parent peptide Cla is broadly effective against both Gram-positive and Gram-negative bacteria [19].

## 5. Conclusions

This study proves that histidine tagging serves as a simple and efficient strategy to enhance the salt tolerance of AMPs, and resolves the critical issue of AMP inactivation during high-salt fermentation using *P. pastoris*. The optimized peptide Cla-H combines strong antifungal activity, rapid fungicidal action, excellent salt resistance and stability. Its crude fermentation product also enables low-cost application and achieves prominent control efficacy against wheat scab and tomato gray mold. This work delivers a promising candidate for developing novel green biofungicides, and establishes a full technical workflow covering molecular modification, microbial expression and in planta evaluation. It thus possesses great theoretical significance and application potential for advancing the use of AMPs in crop disease management.

## Figures and Tables

**Table 1:** The MICs of synthetic Cla and Cla-H against *F. graminearum* and *B. cinerea* under low- (0 mM KCl) and high-salt (100 mM KCl) conditions.

Peptides	MIC (μM) for *F. graminearum*		MIC (μM) for *B. cinerea*	
	Low-Salt	High-Salt	Low-Salt	High-Salt
Chemically synthesized Cla	3.125	nd	12.5	nd
Chemically synthesized Cla-H	3.125	25	12.5	25

Note: ‘nd’ represents ‘not detected’; raw plate images of the resazurin-based microdilution experiments supporting these MIC results are shown in Supplementary Figures S10 and S11. MIC values were determined in triplicates.

## Data Availability

The original contributions presented in this study are included in the article/Supplementary Materials. Further inquiries can be directed to the corresponding authors.

## Supplementary Materials

The following supporting information can be downloaded at https://www.mdpi.com/article/10.3390/biom16091326/s1. Figure S1: PCR-based identification of high-copy *P. pastoris* transformants; Figure S2: Silver-stained Tricine SDS-PAGE analysis of fermentation supernatants from the recombinant *P. pastoris* strains; Figure S3: HPLC analysis of the secreted Cla-H from fermentation supernatant of the engineered yeast strain G-Cla-H; Figure S4: Mass spectra of the biosynthesized Cla-H purified from the G-Cla-H supernatant in comparison with the chemically synthesized Cla-H; Figure S5: Antifungal activity of fermentation supernatants from recombinant *P. pastoris* strains against *B. cinerea* determined using a resazurin colorimetric assay; Figure S6: Representative images showing in vivo antifungal effects of fermentation supernatants from the Cla-H- and GM-H-expressing *P. pastoris* strains against *F. graminearum* in wheat seedlings; Figure S7: Representative images showing in vivo antifungal effects of fermentation supernatants from the Cla-H or GM-H-expressing *P. pastoris* strains along with the empty-vector (EV)-transformed *P. pastoris* against *B. cinerea* on tomato seedlings; Figure S8: Analysis of the antifungal activities of both biosynthetic and chemically synthesized Cla-H peptides against *F. graminearum* spores under low-salt condition (A) and high-salt condition containing 100 mM KCl (B); Figure S9: Analysis of the antifungal activities of both biosynthetic and chemically synthesized Cla-H peptide against *B. cinerea* spores under low-salt condition (A) and high-salt condition containing 100 mM KCl (B); Figure S10: Representative resazurin colorimetric images for determining MICs of Cla H against *F. graminearum* (A) and *B. cinerea* (B) under the low-salt condition; Figure S11: Representative resazurin colorimetric images for determining MICs of Cla-H against *F. graminearum* (A) and *B. cinerea* (B) under the high-salt (100 mM KCl) condition; Figure S12: Comparison of antifungal activities between the Cla-H peptide and the commercial pesticide ‘Jin-mai-tian’ against *F. graminearum* spores; Figure S13: Inhibitory effects of Cla-H on spore germination of *B. cinerea*; Figure S14: Representative SYTOX Green fluorescence micrographs showing the membrane permeability of spores of B. cinerea after GM-H treatment; Figure S15: Representative SYTOX green fluorescence micrographs showing the membrane permeability of spores (A) and hyphae (B) of *B. cinerea* after Cla-H treatment; Figure S16: Representative PI fluorescence micrographs showing time-dependent spore death of *F. graminearum* treated by Cla-H; Figure S17: Representative PI fluorescence micrographs showing time-dependent spore death of *B. cinerea* treated by Cla-H; Table S1: Sequence and physicochemical characteristics of the peptides used in this study.
