# Supplementary material for: Styela clava-Derived Peptide Cla-H Is a Promising Green Biofungicide for Crop Protection
Source: Biomolecules. 2026 Sep 11;16(9):1326. doi: 10.3390/biom16091326 (PMC13604612; doi:10.3390/biom16091326)
Supplement: Supplementary file 1 [file biomolecules-16-01326-s001.zip › biomolecules-4544888-supplementary.pdf]

Supplementary data for

***Styela clava*-derived peptide Cla-H is a promising green biofungicide for crop protection**

Yuliang Xu, Lang Su, Yating Zhang, Changfu Li* and Yansheng Zhang*

Shanghai Key Laboratory of Bio-Energy Crops, School of Life Sciences, Shanghai University, Shanghai 200444, China.

* Corresponding authors:

Yansheng Zhang, zhangys1@shu.edu.cn;

Changfu Li, changfuli@shu.edu.cn

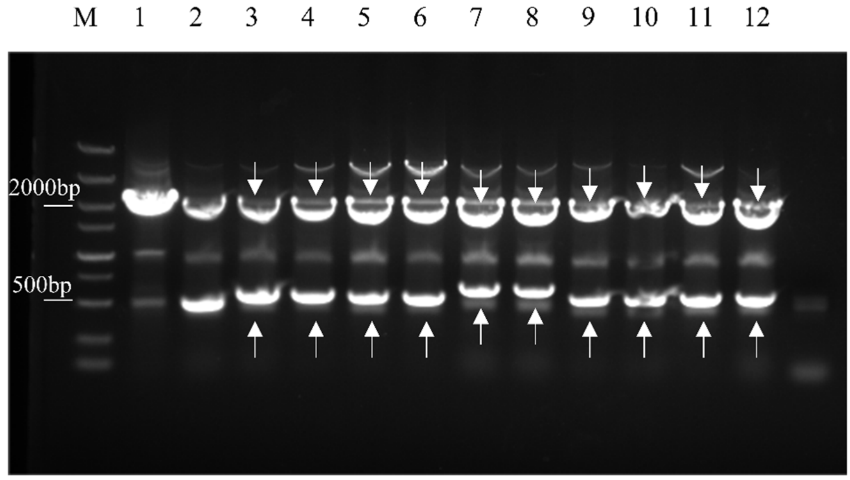

Figure S1. PCR-based identification of high-copy *P. pastoris* transformants.
Lane 1: PCR amplification of the mixture of genomic DNA from wild *P. pastoris* GS115 and empty vector plasmid pPIC9K. Lanes 2–13: PCR products from genomic DNA of strains integrated with empty vector or various peptide-encoding genes, corresponding to G-pPIC9K, G-Ci-H, G-H-Ci, G-GM-H, G-H-GM, G-Zm-H, G-H-Zm, G-Hn-H, G-H-Hn, , G-Cla-H and G-H-Cla, respectively.
Note: The upper white arrowheads indicate the ~2000 bp amplicons from the endogenous *P. pastoris* AOX1 gene; the lower white arrowheads mark PCR products of 480–500 bp, which correspond to the integrated expression cassettes of the target recombinant constructs.

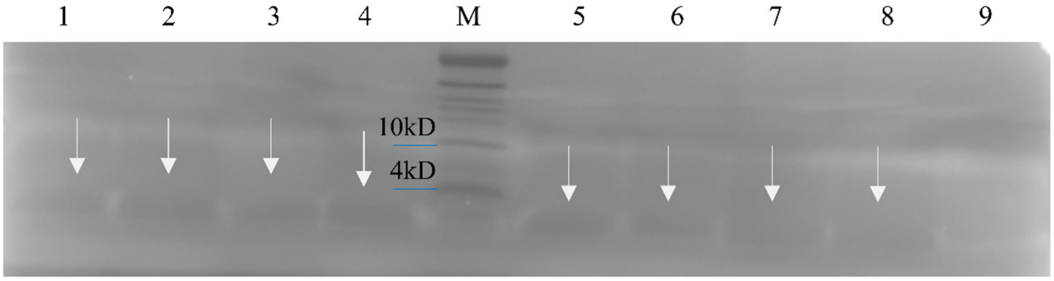

Figure S2. Silver-stained Tricine SDS-PAGE analysis of fermentation supernatants from recombinant *P. pastoris* strains. Lane 1: G-Ci-H; Lane 2: G-H-Ci; Lane 3: G-GM-H; Lane 4: G-H-GM; Lane 5: G-Zm-H; Lane 6: G-H-Zm; Lane 7: G-Cla-H; Lane 8: G-H-Cla; Lane 9: empty-vector control G-pPIC9K. White arrows mark the target recombinant peptides secreted by the engineered strains.

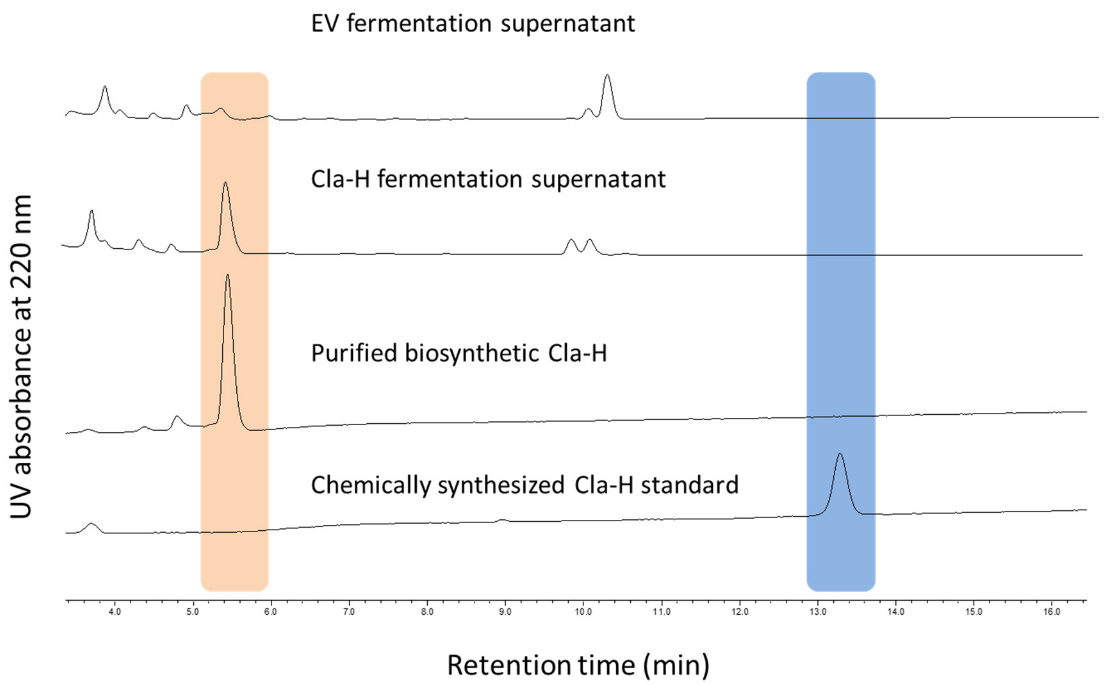

Figure S3. HPLC analysis of the secreted Cla-H from fermentation supernatant of the engineered yeast strain G-Cla-H. Compared with the fermentation supernatant of the empty-vector (EV) transformant, a unique peak was detected in the supernatant of the Cla-H-expressing strain. However, its retention time (orange shaded region, 5.5 min) was markedly different from that of the chemically-synthesized Cla-H standard (blue shaded region, 13.15 min). The biosynthesized Cla-H was further purified and characterized via MS/MS and compared with the chemically synthesized Cla-H standard (mass-spectrometry data shown in Figure S4).

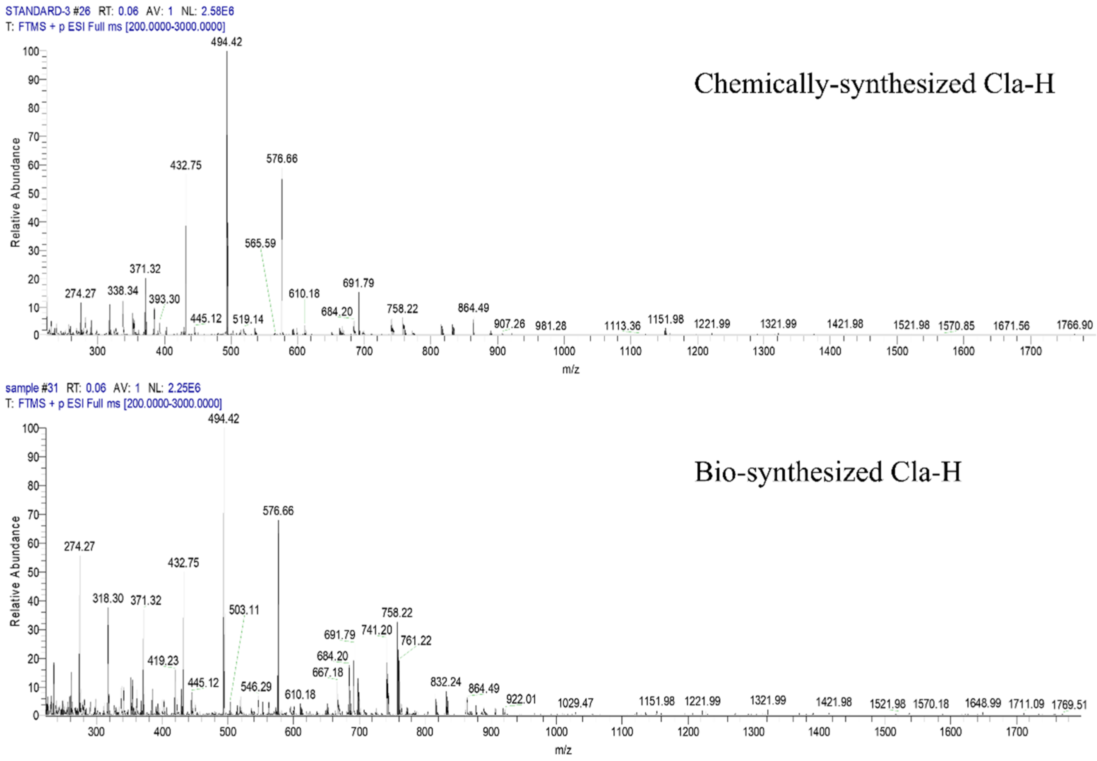

Figure S4. Mass spectra of the biosynthesized Cla-H purified from the G-Cla-H supernatant in comparison with the chemically synthesized Cla-H.

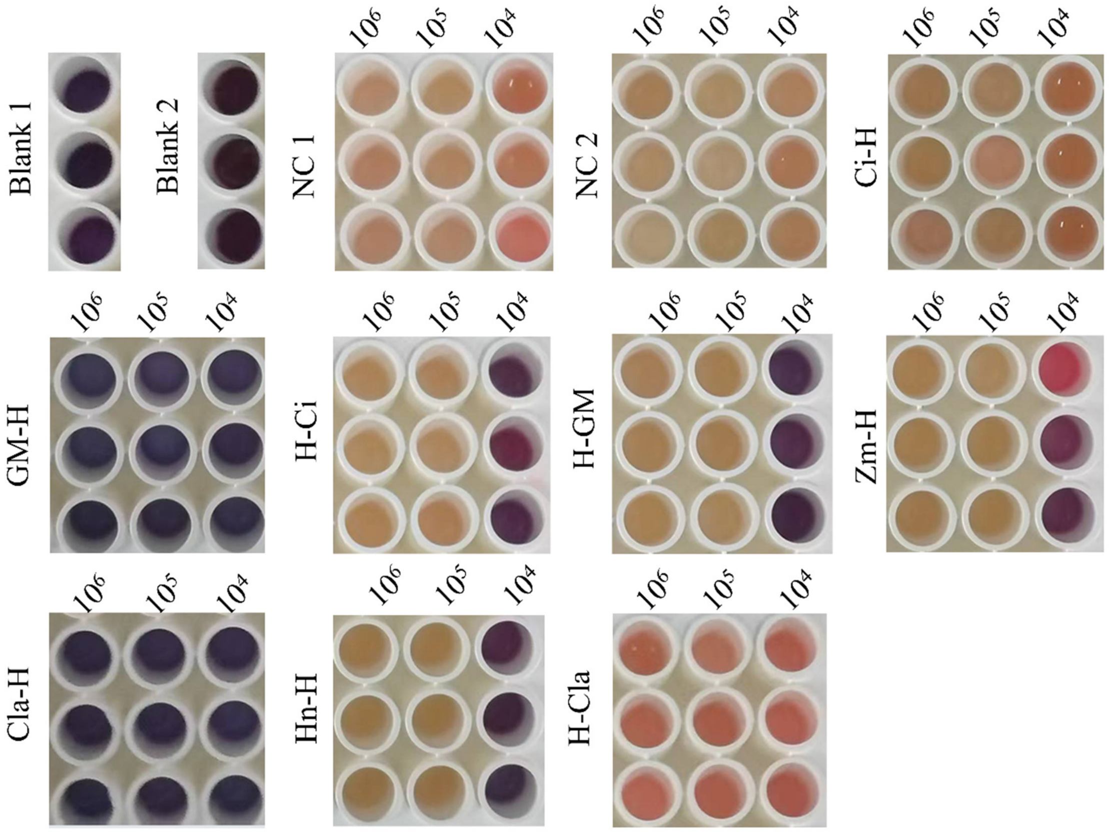

Figure S5. Antifungal activity of fermentation supernatants from recombinant *P. pastoris* strains against *B. cinerea* determined using a resazurin colorimetric assay.

All reaction systems had a final volume of 100 μL. Each system was prepared by mixing double-concentration spore suspension in PDB with an equal volume of test solutions at 1:1 (v/v) ratio. Two blank controls were set up: Blank 1 consisted of PDB mixed with BMMY; Blank 2 was prepared by combining PDB with supernatant from the empty vector-integrated strain. Two negative controls were also included: Negative control 1 (NC 1) contained BMMY and *B. cinerea* spores in PDB; Negative control 2 (NC 2) contained supernatant from the empty vector-integrated strain and *B. cinerea* spores in PDB. Each treatment group was composed of fermentation supernatant from the corresponding recombinant strain and *B. cinerea* spores in PDB. Columns marked with 10^6, 10^5, and 10^4 indicate tests conducted with spore concentration of 10^6, 10^5, and 10^4 sporesmL^{-1}, respectively. The abbreviations beside each nine-well panel represent different engineered antimicrobial peptide variants.

A B

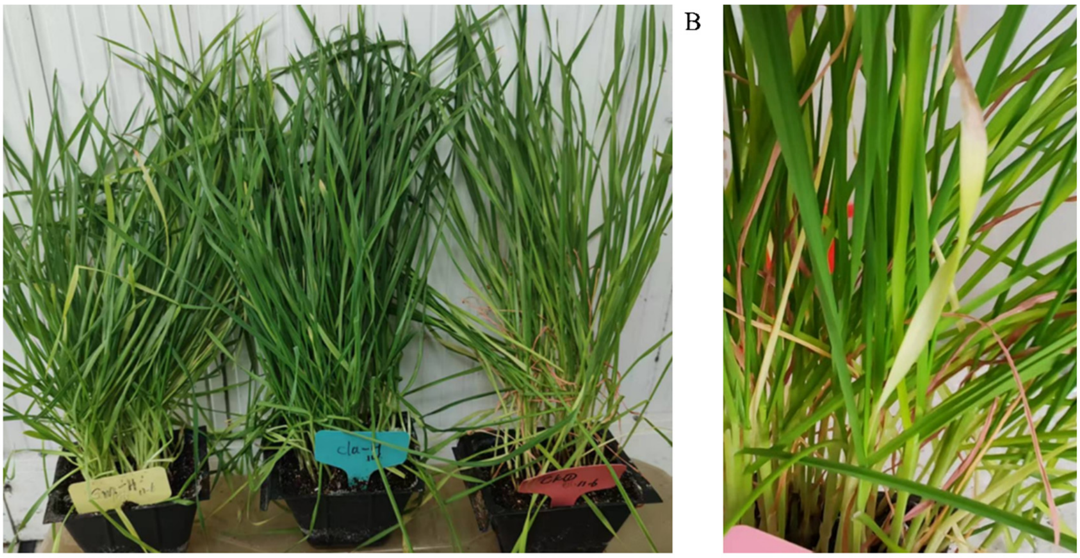

Figure S6. Representative images showing *in vivo* antifungal effects of fermentation supernatants from the Cla-H- and GM-H expressing *P. pastoris* strains against *F. graminearum* in wheat seedlings.

(A) Representative photographs of intact wheat plants subjected to disease-prevention assays with fermentation supernatants from GM-H-, Cla-H-expressing yeast and empty vector (EV)-transformed yeast, respectively; (B) Magnified view of a whole diseased wheat plant from the EV control group.

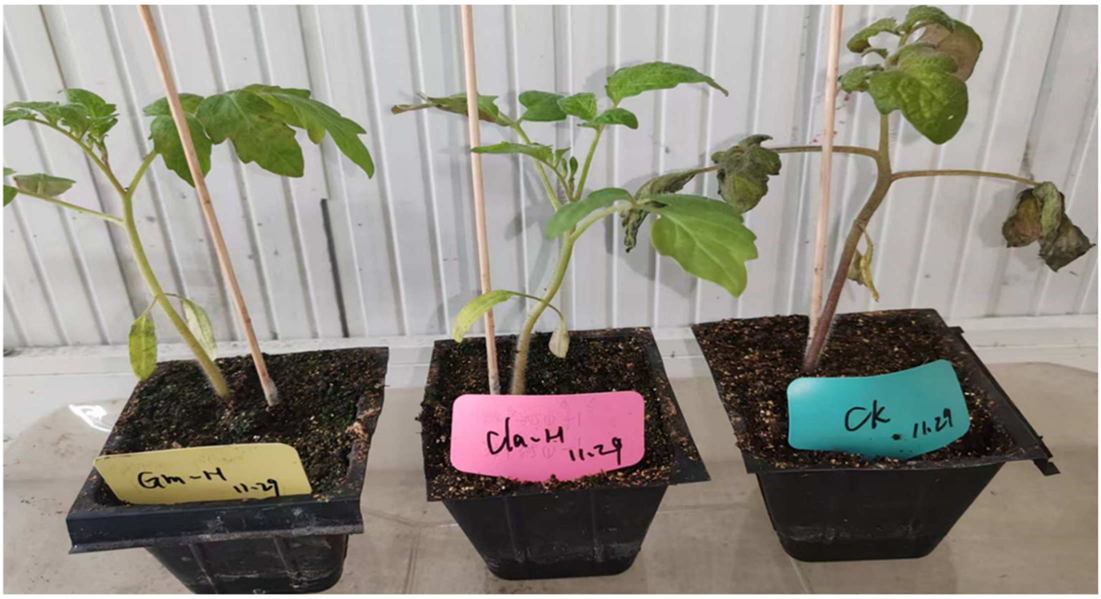

Figure S7. Representative images showing *in vivo* antifungal effects of fermentation supernatants from the Cla-H or GM-H expressing *P. pastoris* strains along with the empty vector (EV)-transformed *P. pastoris* (CK) against *B. cinerea* on tomato seedlings.

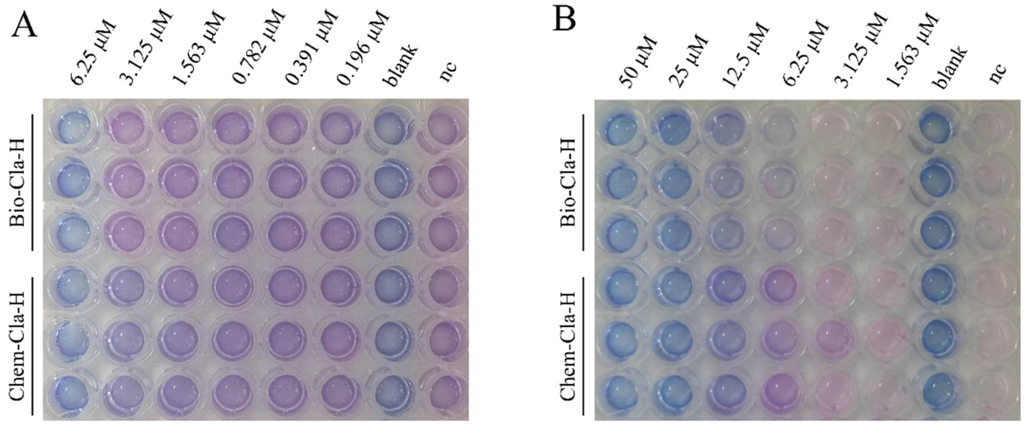

Figure S8. Analysis of the antifungal activities of both biosynthetic and chemically-synthesized Cla-H peptides against *F. graminearum* spores under low-salt condition (A) and high-salt condition containing 100 mM KCl (B). Bio-Cla-H, biosynthetic Cla-H; Chem-Cla-H, chemically-synthesized Cla-H; blank, medium-only control containing 1 × SFM fungal medium plus resazurin; nc (negative control), 1 × SFM fungal medium supplemented with fungal spores (2×10^4 spores/mL) and resazurin; the remaining groups correspond to different concentrations of peptide dissolved in 1 × SFM fungal medium, with fungal spores (2×10^4 spores/mL) and resazurin added.

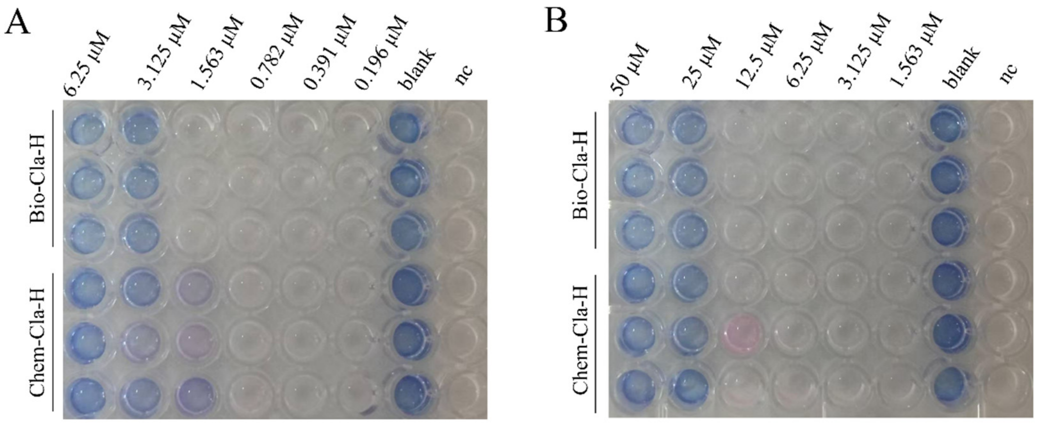

Figure S9. Analysis of the antifungal activities of both biosynthetic and chemically synthesized Cla-H peptide against *B. cinerea* spores under low-salt condition (A) and high-salt condition containing 100 mM KCl (B). Bio-Cla-H, biosynthetic Cla-H; Chem-Cla-H, chemically-synthesized Cla-H; blank, medium-only control containing 1 × SFM fungal medium plus resazurin; nc (negative control), 1 × SFM fungal medium supplemented with fungal spores (2×10^4 spores/mL) and resazurin; the remaining groups correspond to different concentrations of peptide dissolved in 1 × SFM fungal medium, with fungal spores (2×10^4 spores/mL) and resazurin added.

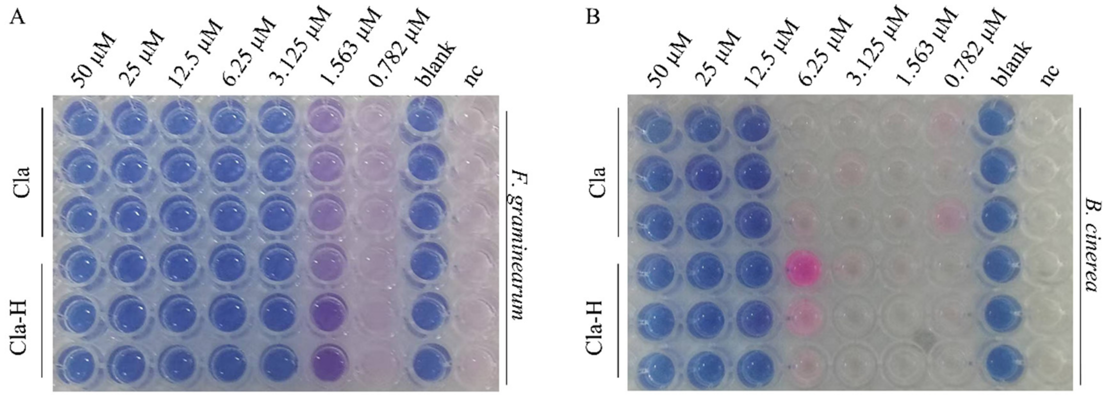

Figure S10. Representative resazurin colorimetric images for determining MICs of chemically synthesized Cla and Cla-H against *F. graminearum* (A) and *B. cinerea* (B) under the low salt condition. blank, medium-only control containing 1 × SFM fungal medium plus resazurin; nc (negative control), 1 × SFM fungal medium supplemented with fungal spores (2 × 10^4 spores/mL) and resazurin; the remaining groups correspond to different concentrations of peptide dissolved in 1 × SFM fungal medium, with fungal spores (2 × 10^4 spores/mL) and resazurin added.

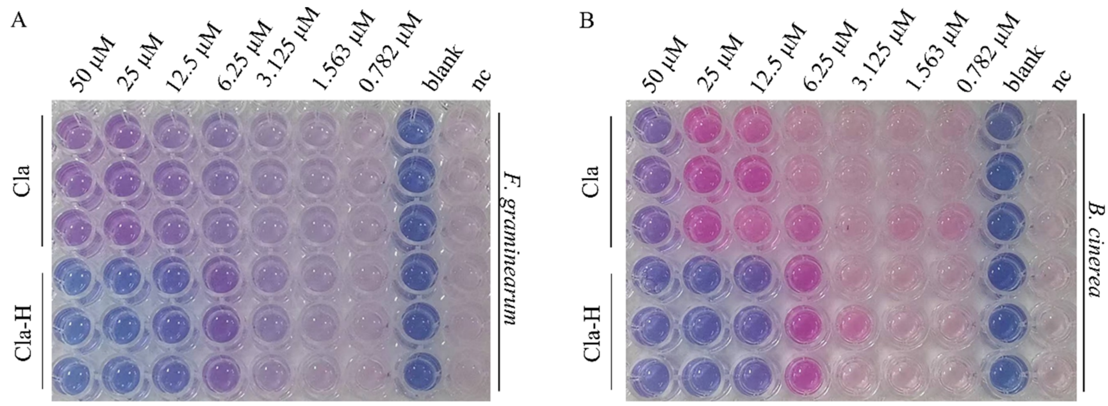

Figure S11. Representative resazurin colorimetric images for determining MICs of chemically synthesized Cla and Cla-H against *F. graminearum* (A) and *B. cinerea* (B) under the high salt condition. blank, medium-only control containing 1 × SFM fungal medium plus resazurin; nc (negative control), 1 × SFM fungal medium supplemented with fungal spores (2 × 10^4 spores/mL) and resazurin; the remaining groups correspond to different concentrations of peptide dissolved in 1 × SFM fungal medium, with fungal spores (2 × 10^4 spores/mL) and resazurin added.

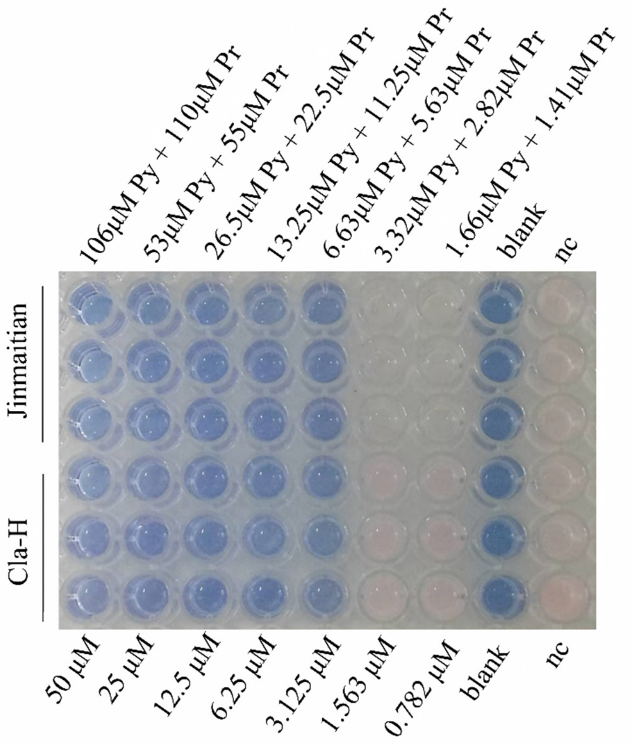

Figure S12. Comparison of antifungal activities between the Cla-H peptide and the commercial pesticide 'Jin-mai-tian' against *F. graminearum* spores. blank, medium-only control containing 1 × SFM fungal medium plus resazurin; nc (negative control), 1 × SFM fungal medium supplemented with fungal spores (2 × 10^4 spores/mL) and resazurin.

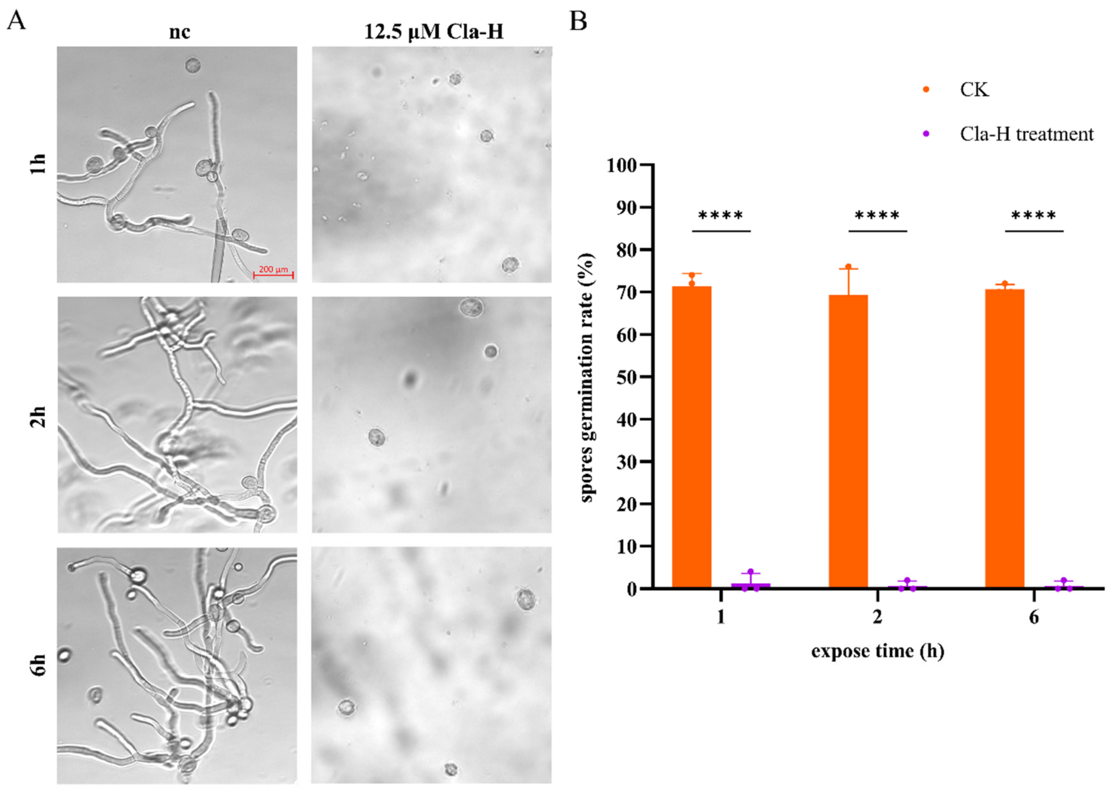

Figure S13. Inhibitory effects of Cla-H on spore germination of *B. cinerea*. Scale bars = 200 μm. Panel A shows representative microscopic images of spore morphology, and Panel B presents statistical data of germination inhibition ratios. **** denotes $P < 0.0001$, representing an extremely significant difference between the compared groups.

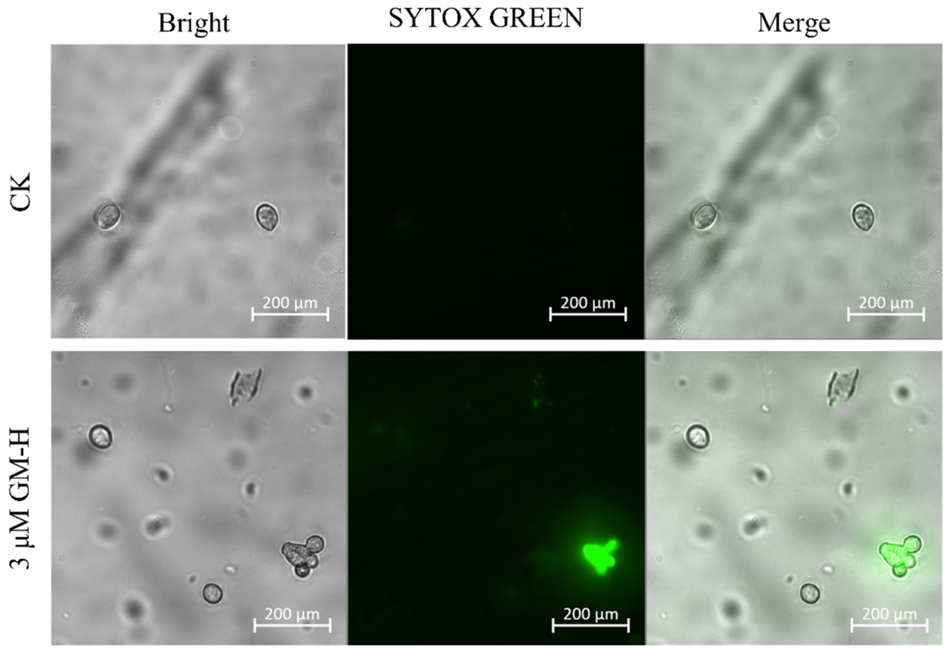

Figure S14. Representative SYTOX Green fluorescence micrographs showing the membrane permeability of spores of *B. cinerea* after GM-H treatment. Scale bars = 200 μm. Images were captured under bright-field, SYTOX Green fluorescence, and merged channels. CK: negative control, representing spores incubated with sterile water without GM-H treatment. Green fluorescence indicates permeabilized cells.

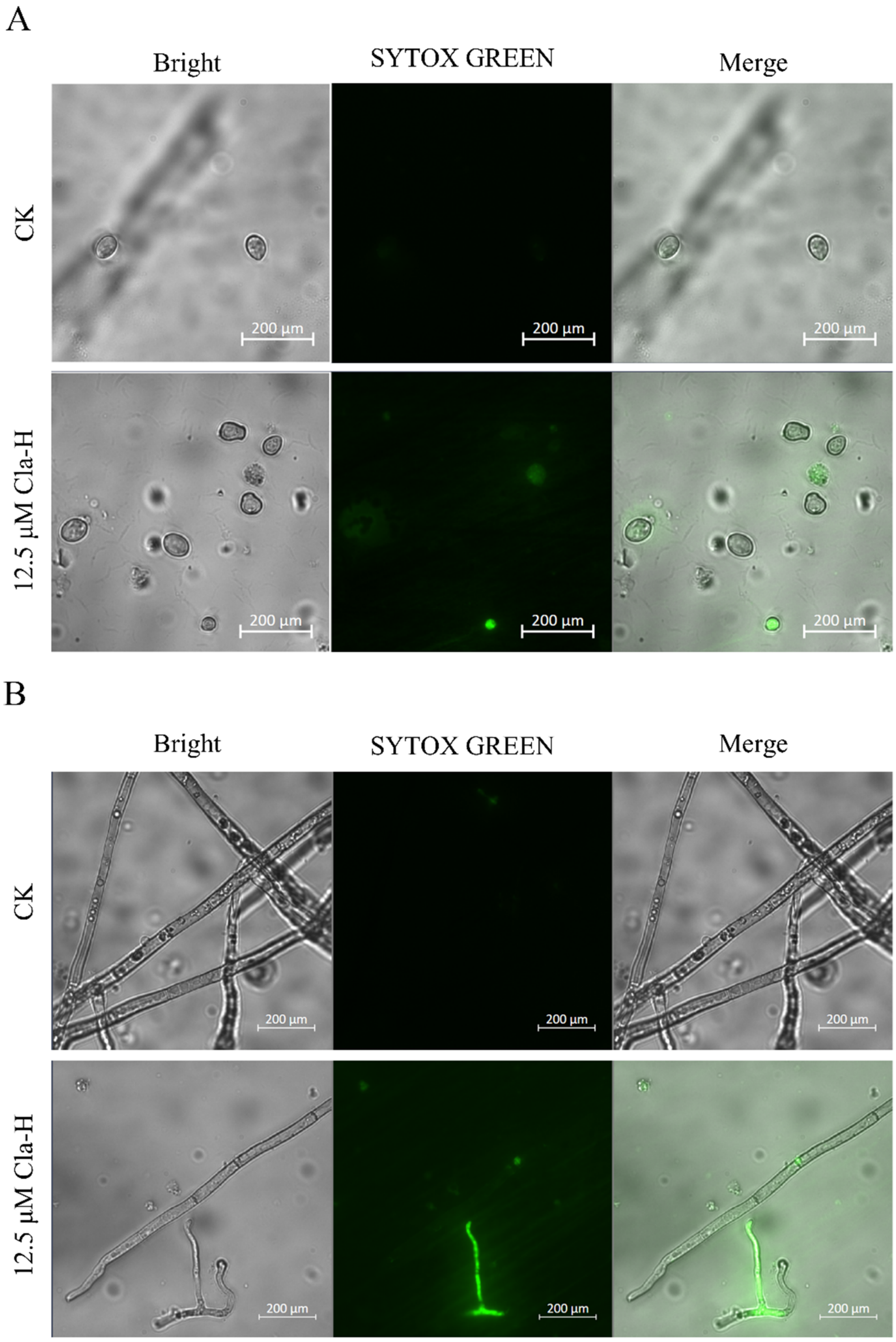

Figure S15. Representative SYTOX Green fluorescence micrographs showing the membrane permeability of spores (A) and hyphae (B) of *B. cinerea* after Cla-H treatment. Scale bars = 200 μm. Images were captured under bright-field, SYTOX Green fluorescence, and merged channels. CK: negative control, representing spores or hyphae incubated with sterile water without Cla-H treatment. Green fluorescence indicates permeabilized cells.

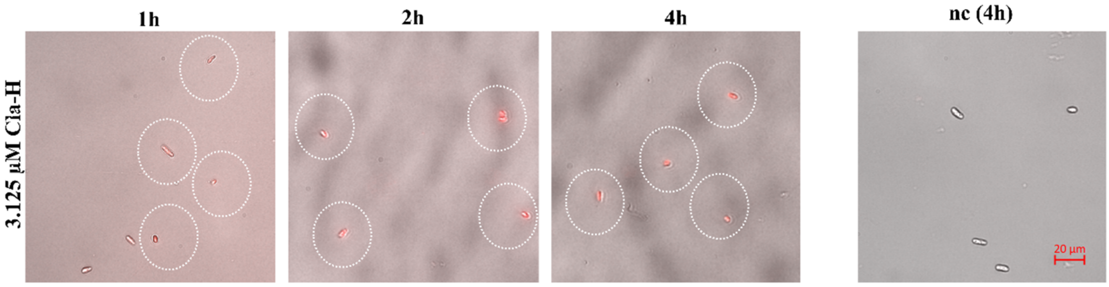

Figure S16. Representative PI fluorescence micrographs showing time-dependent spore death of *F. graminearum* treated by Cla-H. nc: Negative control group, where fungal spores were incubated with sterile water for 4 h. White dotted circles denote PI-stained dead *F. graminearum* spores resulting from Cla-H treatment for 1 h, 2 h and 4 h. Scale bars = 200 µm.

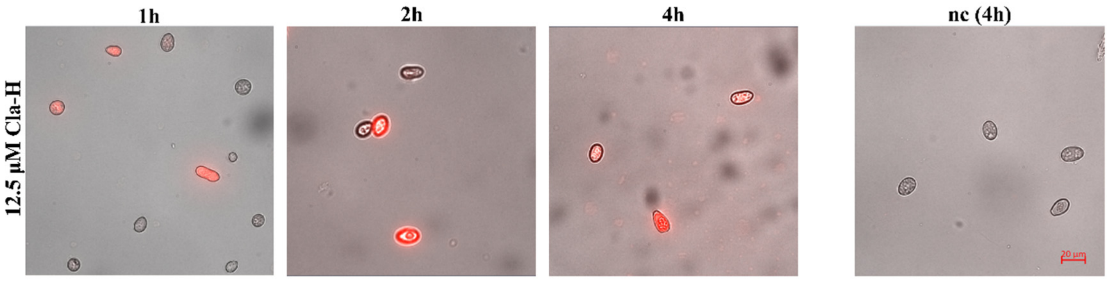

Figure S17. Representative PI fluorescence micrographs showing time-dependent spore death of *B. cinerea* treated by Cla-H. Scale bars = 200 µm. nc: Negative control group, where fungal spores were incubated with sterile water for 4 h. PI-stained red spores denote dead *B. cinerea* spores resulting from Cla-H treatment for 1 h, 2 h and 4 h.

Table S1: Sequence and physicochemical characteristics of the peptides used in this study

Peptides	Species sources	Amino acid sequences	Molecular masses	Net charges	Isoelectric points	References
GMA4CG_V6(GM)	*Medicago truncatula*	GGRCKGFRRRWFWTRIC	2.1856 kDa	6.0	11.83	18
ZmD32(Zm)	*Zea mays*	RTCQSQSHRFRGPCLRRRSN CANVCRTEGFPGG GRCRGFRRRRCFCTTTHC	5.9368 kDa	10.2	11.48	19
Clavanin AK(Cla)	*Styela clava*	VFQFLGKIIKKVGNFVKGFSK VF	2.6312 kDa	5.0	10.60	20
Ci-MAM-A24(Ci)	*Ciona intestinalis*	WRSLGRTLLRLSHALKPLAR RSGW	2.8314 kDa	6.1	12.60	21
HnMc-WP2(Hn)	Chemically synthesized	WKRLKKLIPQQPWIKRKR	2.6312 kDa	8.0	12.32	22
Ci-H	Derived from Ci	WRSLGRTLLRLSHALKPLAR RSGWHHHHHH	3.6542 kDa	6.7	12.60	This study
H-Ci	Derived from Ci	HHHHHHWRSLGRTLLRLSH ALKPLARRSGW	3.6542 kDa	6.7	12.60	This study
GM-H	Derived from GM	GGRCKGFRRRWFWTRICHH HHHH	3.0084 kDa	6.6	11.83	This study
H-GM	Derived from GM	HHHHHHGGRCKGFRRRWF WTRIC	3.0084 kDa	6.6	11.83	This study
Zm-H	Derived from Zm	RTCQSQSHRFRGPCLRRRSN CANVCRTEGFPGGGRCRGF RRRRCFCTTTHCHHHHHH	6.7596 kDa	12.8	11.48	This study
H-Zm	Derived from Zm	HHHHHHRTCQSQSHRFRGP CLRRRSNCANVCRTEGFPG GGRCRGFRRRRCFCTTTHC	6.7596 kDa	12.8	11.48	This study
Hn-H	Derived from Hn	WKRLKKLIPQQPWIKRKRH HHHHH	3.2258 kDa	8.6	12.32	This study
H-Hn	Derived from Hn	HHHHHHWKRLKKLIPQQP WIKRKR	3.2258 kDa	8.6	12.32	This study
Cla-H	Derived from Cla	VFQFLGKIIKKVGNFVKGFSK VFHHHHHH	3.4541 kDa	5.6	10.60	This study
H-Cla	Derived from Cla	HHHHHHVFQFLGKIIKKVG NFVKGFSKVF	3.4541 kDa	5.6	10.60	This study
